# The Impact of Extracurricular Activities on Pre-Service Teacher Professional Development: A Structural Equation Modeling Study

**DOI:** 10.3390/jintelligence13070087

**Published:** 2025-07-17

**Authors:** Funda Uysal

**Affiliations:** Department of Curriculum and Instruction, Faculty of Education, Burdur Mehmet Akif Ersoy University, 15030 Burdur, Turkey; fuysal@mehmetakif.edu.tr

**Keywords:** extracurricular activities, teacher education, self-efficacy, professional interest, self-regulation, teacher–student relationships, social cognitive theory, self-determination theory, self-regulation theory, interpersonal relationships theory

## Abstract

This study investigates the development of cognitive, emotional, and social skills in pre-service teachers through extracurricular activities, addressing 21st century challenges in preparing educators for diverse learning environments. It was hypothesized that extracurricular activities would positively influence cognitive skills (self-efficacy, self-regulation), emotional dimensions (professional interest), social competencies (teacher–student relationships), and academic achievement. This study employed predictive correlational methodology based on an integrated theoretical framework combining Social Cognitive Theory, Self-Determination Theory, Self-Regulation Theory, and Interpersonal Relationships Theory within formal–informal learning contexts. A psychometrically robust instrument (“Scale on the Contribution of Extracurricular Activities to Professional Development”) was developed and validated through exploratory and confirmatory factor analyses, yielding a five-factor structure with strong reliability indicators (Cronbach’s α = 0.91–0.93; CR = 0.816–0.912; AVE = 0.521–0.612). Data from 775 pre-service teachers (71.1% female) across multiple disciplines at a Turkish university were analyzed using structural equation modeling (χ^2^/df = 2.855, RMSEA = 0.049, CFI = 0.93, TLI = 0.92). Results showed that extracurricular participation significantly influenced self-efficacy (β = 0.849), professional interest (β = 0.418), self-regulation (β = 0.191), teacher–student relationships (β = 0.137), and academic achievement (β = 0.167). Notably, an unexpected negative relationship emerged between self-efficacy and academic achievement (β = −0.152). The model demonstrated strong explanatory power for self-efficacy (R^2^ = 72.8%), professional interest (R^2^ = 78.7%), self-regulation (R^2^ = 77.2%), and teacher–student relationships (R^2^ = 63.1%) while explaining only 1.8% of academic achievement variance. This pattern reveals distinct developmental pathways for professional versus academic competencies, leading to a comprehensive practical implications framework supporting multidimensional assessment approaches in teacher education. These findings emphasize the strategic importance of extracurricular activities in teacher education programs and highlight the need for holistic approaches beyond traditional academic metrics, contributing to Sustainable Development Goal 4 by providing empirical evidence for integrating experiential learning opportunities that serve both academic researchers and educational practitioners seeking evidence-based approaches to teacher preparation.

## 1. Introduction

Contemporary teacher education recognizes that professional development extends far beyond formal curriculum, occurring primarily through informal learning contexts where knowledge emerges from experience, reflection, and social interaction (Eraut 2004). From this perspective, extracurricular activities represent essential informal learning environments that enable pre-service teachers to develop professional competencies through authentic, self-directed engagement (Marsick and Watkins 2001).

Teacher professional development encompasses multiple interconnected components that contribute to educator effectiveness. Research identifies several key components of pre-service teacher professional development: (1) pedagogical content knowledge and subject-specific expertise, (2) instructional methodology and teaching strategies, (3) educational psychology and child development, (4) classroom management and behavior guidance, (5) assessment and evaluation methodologies, and (6) supervised field experiences and student teaching (Darling-Hammond et al. 2017; Shulman 1987). These formal education components provide structured educational experiences through systematic curriculum frameworks and predetermined learning sequences that establish foundational competencies essential for teacher certification. However, informal learning—characterized by spontaneous, self-directed experiences occurring outside traditional educational settings—serves as an equally vital complement to formal preparation. As emphasized by Eraut (2004), informal learning occurs through everyday experiences, interactions, and self-directed exploration that fills gaps left by formal education systems. Research demonstrates that experiential learning during early stages of teacher professional development enables educators to observe student outcomes, experience new pedagogical approaches as learners themselves, and adapt implementations in authentic classroom contexts (Melber and Cox-Petersen 2005). The theoretical frameworks guiding this study—Social Cognitive Theory, Self-Determination Theory, and Self-Regulation Theory—apply across both formal and informal learning contexts yet manifest differently in each setting (Bandura 1997; Ryan and Deci 2000; Zimmerman 2000). While acknowledging the foundational importance of formal curriculum components in providing systematic knowledge and skill development, this study focuses specifically on the informal learning dimension through extracurricular activities to address a significant gap in teacher education research that has primarily examined formal curriculum components while neglecting substantial contributions of informal learning experiences to professional development outcomes (Wolf et al. 2021). This comprehensive understanding of formal–informal learning integration provides essential context for examining teacher education effectiveness in contemporary educational environments.

The quality of teacher education is one of the most critical factors determining the effectiveness of education systems (Darling-Hammond 2020). Today, pre-service teachers are expected to possess a variety of pedagogical and professional skills in addition to strong content knowledge (Révai and Guerriero 2017). Within this informal learning framework, these authentic experiences support professional development—which encompasses various frameworks in the teacher education literature (Darling-Hammond and Bransford 2007; Shulman 1987) but is operationalized in this study through five dimensions: extracurricular participation, self-efficacy, professional interest, self-regulation, and faculty relationships—and academic success of pre-service teachers (Wolf et al. 2021).

In the context of Sustainable Development Goals (SDG) 4 (Quality Education), extracurricular activities may have an important role in the professional and academic development of pre-service teachers (UNESCO 2020). Extracurricular activities contribute to the development of pre-service teachers’ leadership skills, cultural competencies, and environmental awareness necessary for sustainable education. Sustainable education, defined as education that develops knowledge, skills, perspectives, and values for addressing social, economic, and environmental challenges (UNESCO 2021), has become central to contemporary teacher education (Lozano et al. 2017). Cebrián and Junyent’s (2015) research emphasizes the importance of informal learning environments in developing sustainability competencies in teacher education. Salah and Houichi (2023) showed that extracurricular activities increase pre-service teachers’ awareness of sustainability and their practical skills. In this context, UNESCO (2020) states that education for sustainable development should not be limited to formal education but should be supported by extracurricular activities. Given the multifaceted nature of key concepts in this study, clarification is essential. Extracurricular activities—broadly defined in the literature as organized learning experiences beyond formal curriculum (Mahoney et al. 2005)—are operationalized in this research as university-based programs, workshops, conferences, and professional development sessions measured through the developed scale items.

Considering the potential of extracurricular activities, these activities can enable pre-service teachers to improve their self-efficacy (Pekin and Bozdoğan 2021; Yilmaz and Lee 2023), professional interest, self-regulation skills, teacher–student relationships (Uzun and Bolat 2023), and academic achievement (Chongcharoen et al. 2024; Yavuzalp and Özdemir 2021). However, the role and effectiveness of extracurricular activities in teacher education has not been systematically examined (Klassen et al. 2020). While existing studies highlight the benefits, more extensive research is needed to explore how these activities can be optimized to fully support the development of pre-service teachers. This is because the existing literature has separately addressed various factors that play a role in pre-service teachers’ professional development and academic achievement, but studies that holistically examine the complex relationships between these factors are limited (Allen et al. 2022). In particular, there is a need for comprehensive research examining the role of extracurricular activities in pre-service teachers’ professional development and academic performance and the psychological mechanisms mediating this role (Zee and Koomen 2016).

**Figure 1:**
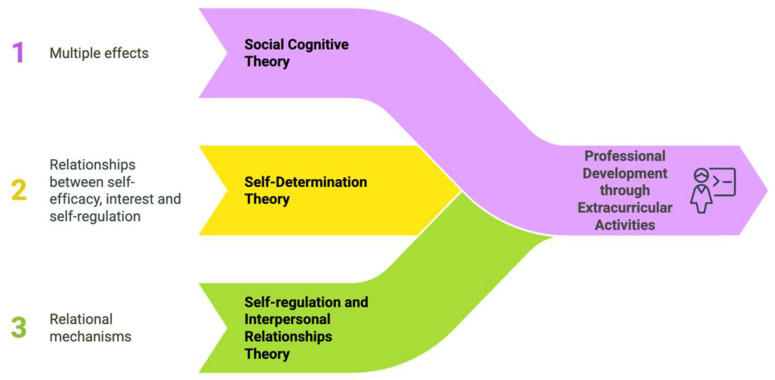
The theoretical framework of this study.

In line with the aforementioned needs, this study examines the role of extracurricular activities in the professional development of pre-service teachers through three main theoretical frameworks, as seen in Figure 1.

The selection of this theoretical framework responds to the need for a comprehensive understanding of how informal learning contexts influence teacher development. While numerous theories could explain aspects of this phenomenon—such as situated learning theory (Lave and Wenger 1991) or transformative learning theory (Mezirow 1991)—this study adopts three complementary frameworks that together address the cognitive, motivational, and relational dimensions of professional development. Social Cognitive Theory provides essential insights into experiential learning and self-efficacy development, Self-Determination Theory explains motivational mechanisms underlying professional interest, and Self-Regulation/Interpersonal Relationships Theories address behavioral and social outcomes. This integrated approach ensures conceptual clarity while capturing the multifaceted nature of teacher professional development within informal learning contexts.

As illustrated in Figure 1, the theoretical framework demonstrates how three complementary theories guide this study’s structure. First, Social Cognitive Theory (Bandura 1986) provides the foundation for examining multiple effects of extracurricular activities on pre-service teachers. Second, Self-Determination Theory (Ryan and Deci 2000) explains the relationships between self-efficacy, professional interest, and self-regulation. Finally, the Self-Regulation and Interpersonal Relationships Theories (Zimmerman 2000; Reis and Shaver 1988) illuminate relational mechanisms in professional development. Together, these frameworks provide the theoretical basis for testing the ten hypotheses of this study, creating a comprehensive model for understanding how extracurricular activities contribute to teacher development.

The integration of these theoretical frameworks reflects the complex, interconnected nature of teacher professional development. Social Cognitive Theory serves as the foundational framework, explaining how extracurricular activities provide direct experience that enhances self-efficacy (Bandura 1997). This enhanced self-efficacy then feeds into Self-Determination Theory’s mechanisms, where fulfillment of competence needs leads to increased intrinsic motivation and professional interest (Ryan and Deci 2000). Finally, the Self-Regulation and Interpersonal Relationships Theories explain how this motivation translates into concrete behavioral changes—improved self-regulation strategies and enhanced relationship-building capacities (Zimmerman 2000; Reis and Shaver 1988). This theoretical synthesis provides a comprehensive understanding of how extracurricular activities contribute to teacher development across cognitive, motivational, and behavioral dimensions.

### 1.1. Social Cognitive Theory and Multiple Effects of Extracurricular Activities

Social Cognitive Theory (Bandura 1986) provides an important theoretical framework for pre-service teachers’ professional development. Recent meta-analyses strongly support the validity of this theory in teacher education (Zee and Koomen 2016). Direct experience opportunities such as extracurricular activities play a critical role in the development of pre-service teachers’ self-efficacy beliefs (Cai et al. 2022). Systematic reviews show that extracurricular activities provide pre-service teachers with experiential learning opportunities beyond their formal education programs to develop their professional skills and strengthen their self-efficacy beliefs (Darling-Hammond et al. 2020). This theoretical foundation supports the hypothesis (H_1_) that participation in extracurricular activities will positively affect pre-service teachers’ self-efficacy.

From the perspective of Social Cognitive Theory, extracurricular activities can also play an important role in the development of pre-service teachers’ professional interests. König et al. (2017) emphasize the importance of various learning opportunities in the professional development of pre-service teachers. In this context, extracurricular activities may increase professional interest by providing opportunities to experience and explore different aspects of the teaching profession. Ginosyan et al. (2020) found that students participating in extracurricular activities increased their motivation and also deepened their understanding of the teaching profession by experiencing different roles in the educational environment. This is necessary for the development of professional interest in teaching. Jæger and Møllegaard (2017) discuss how participation in cultural and extracurricular activities enhances creativity and academic competence and how these are beneficial for educational success. This suggests that the benefits of extracurricular activities in enhancing professional interest are valid in a variety of educational settings. This theoretical framework supports the hypothesis (H_2_) that participation in extracurricular activities will increase interest in the teaching profession.

In terms of self-regulation skills, Social Cognitive Theory emphasizes that individuals plan, monitor, and evaluate their own learning processes (Zimmerman and Schunk 2013). Recent research emphasizes that extracurricular activities contribute to the development of self-regulation skills by providing unstructured learning environments (Dignath and Büttner 2018). Meta-analytic findings suggest that this effect is particularly strong at the higher education level (Dinsmore and Wilson 2016). This theoretical basis supports the hypothesis (H_3_) that extracurricular activities will positively affect self-regulation skills.

In the dimension of teacher–student relationships, the theory emphasizes the critical role of social interactions in learning and development (Forneris et al. 2014). Extracurricular activities offer pre-service teachers the opportunity to practice interacting with diverse groups of students (Alhasov et al. 2020; Lubis and Mahariah 2024). Systematic reviews have shown that these experiences determine the quality of future teacher–student relationships (McGrath and Van Bergen 2015). This theoretical framework supports the hypothesis (H_4_) that extracurricular activities will improve the quality of teacher–student relationships.

Extracurricular activities are considered a very important component in increasing academic achievement supported by Social Cognitive Theory. Social Cognitive Theory emphasizes that academic achievement is influenced by cognitive abilities, self-efficacy beliefs, and social interactions (Bandura 1997). Recent studies underline the impact of social interaction on academic achievement. For example, Zander et al. (2018) emphasize the importance of academic and social networks in enhancing students’ academic integration and self-efficacy. Cross-cultural research also supports this. For example, Blansky et al. (2013) show how a student’s academic performance can be positively influenced by their immediate social environment, pointing to a phenomenon similar to social contagion, where academic success spreads within peer networks. This theoretical foundation supports the hypothesis (H_5_) that there is a positive correlation between extracurricular activities and academic achievement.

While Social Cognitive Theory operates across both formal and informal learning contexts in teacher education, this study focuses specifically on informal learning through extracurricular activities to address the underexplored dimension of teacher professional development. In formal curriculum settings, Social Cognitive Theory manifests through structured modeling of teaching practices, supervised field experiences, and systematic feedback mechanisms that build self-efficacy through guided mastery experiences (Darling-Hammond et al. 2020). These formal applications provide essential foundational knowledge and initial skill development through predetermined learning sequences that include classroom observations, mentor-guided practice sessions, and standardized evaluation procedures designed to ensure competency development.

However, informal learning contexts—particularly extracurricular activities—offer unique opportunities for authentic application of Social Cognitive Theory principles. As noted by Eraut (2004), informal learning environments provide spontaneous opportunities for observational learning that cannot be fully replicated in formal settings. Research on teacher professional development emphasizes that informal learning through workplace experiences, collegial interaction, and learning by doing represent essential components of continuous professional development that complement formal training (Eraut 2004). Unlike formal curriculum constraints, extracurricular settings provide flexible environments where pre-service teachers can engage in spontaneous observational learning, experiment with diverse teaching approaches, and develop self-efficacy through self-directed practice. This informal dimension complements formal learning by enabling real-world application of theoretical knowledge, peer-to-peer modeling, and autonomous skill development that formal curricula cannot fully accommodate.

**Figure 2:**
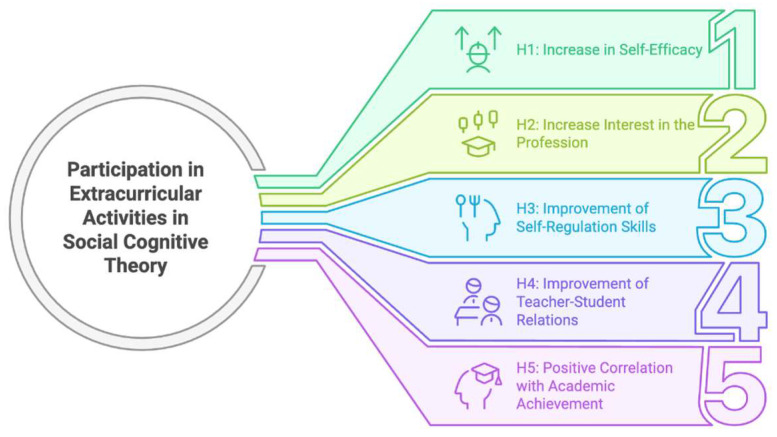
Multiple effects of participation in extracurricular activities in the context of Social Cognitive Theory.

Figure 2 summarizes the multiple effects of participation in extracurricular activities according to the Social Cognitive Theory in the context of the hypotheses.

As seen in Figure 2, according to the hypotheses, extracurricular activities positively affect pre-service teachers’ self-efficacy, professional interest, self-regulation skills, and quality of student relationships and show a positive relationship with their academic achievement.

### 1.2. Self-Determination Theory: Relationships Between Self-Efficacy, Professional Interest, and Self-Regulation

Self-Determination Theory (Ryan and Deci 2000) identifies three basic psychological needs essential for optimal motivation and well-being: autonomy (feeling volitional and self-directed), competence (experiencing mastery and achievement), and relatedness (feeling connected to others). These needs function as universal psychological nutrients that, when satisfied, promote intrinsic motivation, engagement, and psychological well-being (Deci and Ryan 2000).

The concept of “sense of achievement” represents a core component of competence need satisfaction, encompassing both the psychological experience of mastery and the behavioral outcomes that reflect successful performance. When individuals experience competence through successful task completion, skill development, or goal attainment, they develop stronger intrinsic motivation and sustained engagement with the activity domain (Vansteenkiste et al. 2020).

Self-Determination Theory (Ryan and Deci 2000) provides a powerful theoretical framework that explains the relationships between individuals’ intrinsic motivations and psychological needs. Recent research in the context of teacher education emphasizes the role of self-efficacy beliefs in shaping professional interest (Li 2024; Richardson et al. 2014). Kaleli’s (2020) research shows that those with high self-efficacy are more likely to demonstrate positive attitudes and interest in teaching, leading to better academic and professional outcomes. This finding is also supported by Bardach et al. (2021). These findings support the hypothesis (H_6_) that high self-efficacy is associated with increased interest in the teaching profession.

From the perspective of Self-Determination Theory, the relationship between professional interest and self-regulation skills is explained by intrinsic motivation and autonomous learning processes (Vansteenkiste et al. 2020). Strong interest in the teaching profession may be positively related to the use of effective self-regulation strategies (Martin and Collie 2019). Tikhomirova et al. (2021) document how sociocultural conditions shape teachers’ self-regulatory practices, which in turn affect their academic interest and professional commitment in different countries. This broad support reinforces the idea that effective self-regulation is a universal strategy that increases teachers’ commitment to their roles. Recent longitudinal studies also document that professional interest and self-regulation skills are mutually reinforcing (Wolters and Hussain 2015). These theoretical and empirical foundations support the hypothesis (H_9_) that a strong interest in teaching is positively related to effective self-regulation skills.

Supporting autonomous sources of motivation during pre-service teachers’ professional development process contributes to the sustainable development of both self-regulation skills and professional engagement (Pelletier and Rocchi 2016). Meta-analytic findings show that autonomy-supportive learning environments strengthen pre-service teachers’ self-regulation skills and professional engagement (Reeve and Cheon 2021).

Self-Determination Theory’s principles of autonomy, competence, and relatedness function across both formal curriculum and informal learning contexts yet manifest differently in each setting. Formal teacher education programs address these needs through structured choice within curriculum requirements, competency-based progression systems, and collaborative learning experiences designed to fulfill relatedness needs (Reeve and Cheon 2021). These formal applications provide systematic frameworks for motivation development within predetermined educational pathways that include course selections within degree requirements, scaffolded skill-building sequences, and structured peer learning activities that ensure comprehensive professional preparation while maintaining program coherence and quality standards.

Extracurricular activities, as informal learning environments, offer distinct opportunities for authentic Self-Determination Theory application. Research demonstrates that when professional development activities fulfill the basic psychological needs of autonomy, competence, and relatedness, they significantly increase teachers’ motivation and psychological well-being (Pelletier and Rocchi 2016). Unlike formal curriculum constraints, these activities provide genuine autonomy through voluntary participation and self-directed exploration of professional interests without predetermined outcomes or mandated assessments. Competence development occurs through authentic challenges and real-world problem-solving rather than prescribed academic tasks, while relatedness needs are fulfilled through organic professional relationships that develop through shared interests and collaborative engagement. This study’s focus on extracurricular activities specifically examines how Self-Determination Theory operates when pre-service teachers engage in voluntary, autonomous professional development beyond formal requirements.

In this study, SDT’s three basic needs are operationalized through specific constructs: professional interest as an indicator of intrinsic motivation reflecting autonomy, self-efficacy as a reflection of competence need satisfaction, and teacher–student relationships as a measure of relatedness. While academic achievement constitutes a behavioral outcome that may reflect competence need fulfillment, self-efficacy represents the psychological process underlying competence experiences. The decision to examine academic achievement through Social Cognitive Theory (H_5_) and Self-Regulation Theory (H_10_) frameworks, rather than incorporating it directly into SDT-derived hypotheses (H_6_, H_9_), reflects this theoretical positioning that distinguishes between psychological processes (competence need satisfaction through self-efficacy) and behavioral outcomes (academic performance) within the motivation literature.

Within the context of extracurricular activities, Self-Determination Theory provides valuable insights into how these informal learning environments can enhance pre-service teachers’ intrinsic motivation and professional interest. Research demonstrates that when professional development activities fulfill the basic psychological needs of autonomy, competence, and relatedness, they significantly increase teachers’ motivation and psychological well-being (Hortigüela-Alcalá et al. 2020; Pelletier and Rocchi 2016), as reflected in Hypotheses 6 and 9 of this study.

**Figure 3:**
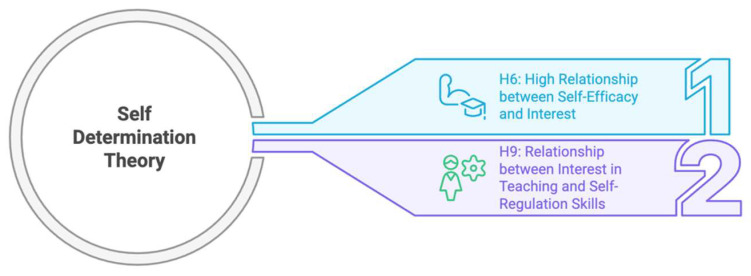
Relationships between self-efficacy, professional interest, and self-regulation in the context of Self-Determination Theory.

Figure 3 summarizes the relationships between self-efficacy, professional interest, and self-regulation according to Self-Determination Theory in the context of the hypotheses.

As seen in Figure 3, the two hypotheses presented within the framework of Self-Determination Theory suggest that high self-efficacy increases interest in the teaching profession and that strong interest in teaching is positively related to effective self-regulation skills.

### 1.3. Self-Regulation and Interpersonal Relationships Theories: Relational Mechanisms in Pre-Service Teachers’ Professional Development

Self-Regulation Theory provides a comprehensive theoretical framework that explains the capacity of individuals to manage and control their own learning processes (Zimmerman and Schunk 2013). Recent research in teacher education emphasizes the role of self-efficacy beliefs in the development of self-regulation skills (Panadero et al. 2019). For example, Mauraji and Wiyarsi (2021) emphasized that pre-service teachers with high levels of self-efficacy showed a greater tendency towards self-regulated learning strategies. These findings provide a strong theoretical basis supporting the hypothesis (H_7_) that high self-efficacy levels positively affect self-regulation skills.

From the perspective of interpersonal relations theory, the link between self-regulation skills and teacher–student relationships is examined within the framework of interactional competence. Reis and Shaver’s (1988) interpersonal relations theory emphasizes the critical role of factors such as regulation of emotions, openness, and sensitivity in the development of interpersonal processes. This theory suggests that individuals’ capacity to regulate their emotions and behaviors is one of the main mechanisms that determine the quality of their relationships. Systematic reviews reveal that pre-service teachers with strong self-regulation skills communicate more effectively with students and develop more supportive relationships (Aldrup et al. 2020). The interpersonal processes of openness and sensitivity emphasized by Reis and Shaver (1988) are important factors that shape the quality of teacher–student relationships. Longitudinal research findings by Perry et al. (2018) also emphasize that social support and self-regulation help to establish and maintain teacher–student relationships. This suggests that as teachers provide sustained support, students’ self-regulation improves, thereby improving their relationships. These theoretical and empirical findings support the hypothesis (H_8_) that better self-regulation skills will lead to improved teacher–student relationships.

From a social cognitive learning perspective, teacher–student relationships can play a mediating role in the relationships between self-efficacy and academic achievement (Roorda et al. 2019). Recent meta-analyses suggest that positive teacher–student relationships may function as a mechanism to support both self-efficacy and academic achievement (Lei et al. 2018). In line with Reis and Shaver’s (1988) model of interpersonal relationships, the quality of interactions between teachers and students is considered as a mediating factor shaping knowledge transfer and learning processes. A study by Doménech-Betoret et al. (2017) also found that self-efficacy influences relational dynamics with teachers, which in turn positively affects academic achievement. This theoretical framework provides a basis to support the hypothesis (H_10_) that teacher–student relationships will mediate the relationship between self-efficacy and academic achievement.

The Self-Regulation and Interpersonal Relationships Theories operate within both formal curriculum and informal learning contexts, each offering distinct developmental opportunities. Formal teacher education provides structured frameworks for developing self-regulation through planned reflection activities, systematic goal-setting exercises, and guided metacognitive practices embedded within coursework (Panadero et al. 2019). These formal approaches include structured reflection assignments, portfolio development processes, and systematic feedback cycles that help pre-service teachers develop metacognitive awareness and self-monitoring capabilities within supportive academic frameworks. Interpersonal relationships in formal contexts develop through assigned partnerships, structured peer feedback systems, and supervised mentoring relationships designed to meet specific learning objectives while ensuring professional growth and competency development.

Informal learning through extracurricular activities provides complementary opportunities for authentic self-regulation and relationship development. Research shows that practical experiences and mentoring relationships help teacher candidates develop metacognitive skills essential for effective teaching practice (Kramarski and Michalsky 2009). These settings enable spontaneous self-assessment, autonomous goal-setting, and natural relationship-building that cannot be fully replicated in formal academic contexts where assessment requirements and structured interactions may limit authentic professional relationship development. Pre-service teachers develop self-regulation skills through managing voluntary commitments, adapting to unexpected challenges, and self-directing their learning experiences without external mandates or predetermined success criteria. This study’s examination of extracurricular activities specifically investigates how these theories manifest in authentic, self-directed professional development contexts that complement formal curriculum components.

The application of these theories to extracurricular activities reveals how informal learning contexts can enhance pre-service teachers’ self-regulation capacities and relationship-building skills. Research shows that practical experiences and mentoring relationships help teacher candidates develop metacognitive skills essential for effective teaching practice (Aldossary et al. 2025; Kramarski and Michalsky 2009). Through extracurricular settings, pre-service teachers develop both the self-regulatory competencies necessary for professional growth and the interpersonal skills that facilitate meaningful connections with faculty mentors, as examined in Hypotheses 7, 8, and 10.

**Figure 4:**
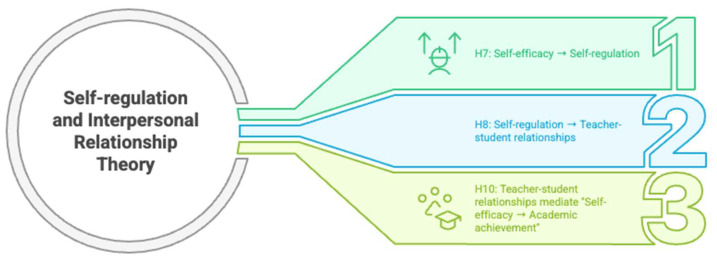
Main and mediating effects in the Self-Regulation and Interpersonal Relationships Theory framework (H_7_, H_8_, H_10_).

Figure 4 summarizes the relational mechanisms in the professional development of pre-service teachers in the context of the Self-Regulation and Interpersonal Relationships Theories in the context of hypotheses.

As illustrated in Figure 4, the Self-Regulation and Interpersonal Relationships Theory framework shows three key relationships. Main effects include self-efficacy positively affecting self-regulation skills (H_7_) and self-regulation skills improving teacher–student relationships (H_8_). The mediating effect is teacher–student relationships mediating the relationship between self-efficacy and academic achievement (H_10_). To clarify a key construct, “teacher–student relationships” in this study specifically refers to relationships between pre-service teachers and university faculty members, as distinguished from relationships with K–12 students. This construct is measured through scale items focusing on mentor–mentee dynamics within the university context.

The complex network of relationships between extracurricular activities, self-efficacy, professional interest, self-regulation skills, and teacher–student relationships in pre-service teachers’ professional development needs to be examined in the light of the theoretical frameworks presented above. Social Cognitive Theory, Self-Determination Theory, and Self-Regulation and Interpersonal Relationships Theories provide a holistic theoretical basis for understanding these relationships. In this context, understanding the role of extracurricular activities in pre-service teachers’ professional development and the psychological mechanisms mediating this role is critical for developing effective teacher education programs.

The purpose of this study is to examine the effects of pre-service teachers’ participation in extracurricular activities on their self-efficacy toward teaching, professional interest in teaching, self-regulation skills, teacher–student relationships, and academic achievement. In line with this general purpose, the following hypotheses were tested:

**Hypothesis** **1 (H1).** *Participation in extracurricular activities positively affects pre-service teachers’ self-efficacy.*

**Hypothesis** **2 (H2).** *Participation in extracurricular activities increases pre-service teachers’ professional interest in teaching.*

**Hypothesis** **3 (H3).** *Participation in extracurricular activities positively affects self-regulation skills.*

**Hypothesis** **4 (H4).** *Participation in extracurricular activities improves the quality of teacher–student relationships.*

**Hypothesis** **5 (H5).** *There is a positive correlation between participation in extracurricular activities and academic achievement.*

**Hypothesis** **6 (H6).** *High self-efficacy is associated with increased interest in the teaching profession.*

**Hypothesis** **7 (H7).** *High self-efficacy levels positively affect self-regulation skills.*

**Hypothesis** **8 (H8).** *Better self-regulation skills lead to improved teacher–student relationships.*

**Hypothesis** **9 (H9).** *A strong interest in teaching is positively related to effective self-regulation skills.*

**Hypothesis** **10 (H10).** *Teacher–student relationships mediate the relationship between self-efficacy and academic achievement.*

**Figure 5:**
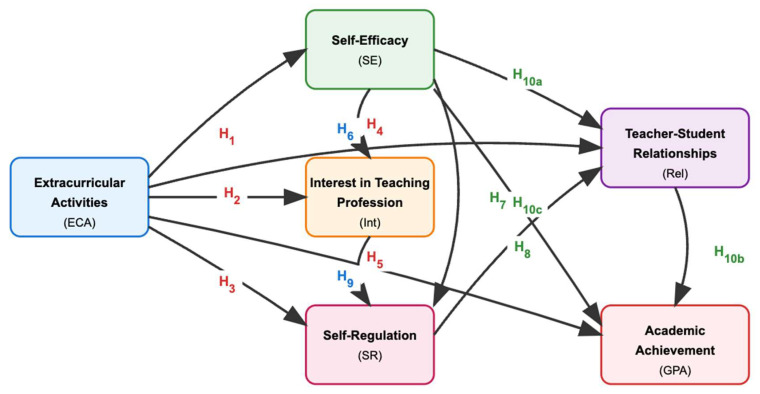
Hypothetical model with hypothesis labels.

Figure 5 provides a comprehensive visual representation of all the hypotheses presented above and their relationships within the integrated theoretical framework.

As shown in Figure 5, the model proposes relationships from three integrated theoretical frameworks: Social Cognitive Theory (H_1_–H_5_), which examines direct effects of extracurricular activities on all outcome variables; Self-Determination Theory (H_6_, H_9_), which focuses on intrinsic motivation and interest development; and the Self-Regulation and Interpersonal Relationships Theories (H_7_, H_8_, H_10_), which explore self-regulation processes and relationship dynamics. The color-coded hypothesis labels facilitate identification of theoretical foundations for each proposed relationship. The model includes both direct effects (single arrows) and complex mediation pathways, with H_10_ representing multiple interconnected relationships involving teacher–student relationships as a mediator.

In order to test the hypotheses presented above, firstly, a valid and reliable measurement tool was developed to measure the contribution of extracurricular activities to professional development, and then structural equation modeling was used to test the direct and indirect relationships between variables simultaneously. This approach allows for a holistic examination of the complex web of relationships among the variables that play a role in the professional development of pre-service teachers.

## 2. Materials and Methods

This study was designed in a predictive correlational research design. This design aims to examine the relationships between variables and determine the level of prediction of one variable by other variables (Creswell 2015; Fraenkel et al. 2006). In the current study, since its aim was to determine the extent to which participation in extracurricular activities predicted pre-service teachers’ self-efficacy, professional interest, self-regulation skills, and teacher–student relationships, this design was preferred. In addition, structural equation modeling, which allows testing direct and indirect relationships between variables simultaneously, was used (Kline 2023). This approach allows the examination of complex relationship patterns in a holistic manner and the testing of theoretical models with empirical data (Hair et al. 2019).

**Figure 6:**
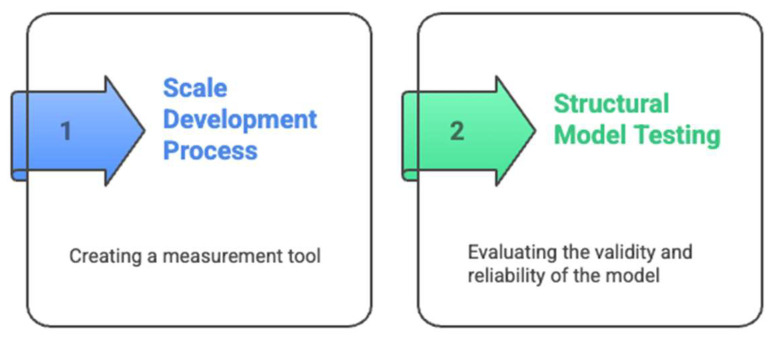
Stages of the research.

This study was designed with a coherent theoretical framework where both the research hypotheses and the scale development process were grounded in the same theoretical foundations: Social Cognitive Theory (Bandura 1986), Self-Determination Theory (Ryan and Deci 2000), Self-Regulation Theory (Zimmerman 2000), and Interpersonal Relationships Theory (Reis and Shaver 1988). This alignment ensures theoretical consistency throughout the research. Taking this context into account, Figure 6 shows the stages of the research.

As seen in Figure 6, the research was conducted in two main stages. In the first main stage in the context of the theoretical framework, a valid and reliable instrument measuring participation in extracurricular activities was developed. In the scale development process, exploratory and confirmatory factor analyses were conducted on different samples (Brown 2015). In the second main stage, the proposed structural model was tested. Prior to model testing, the psychometric properties of the measurement tool were re-examined with confirmatory factor analysis and the validity of the measurement model was confirmed (Byrne 2011).

This research design provides the necessary methodological infrastructure to understand the role of extracurricular activities in pre-service teachers’ professional development. Predictive correlational design and structural equation modeling approach were preferred because they allow testing complex relationships between variables within a single model and account for measurement error (Alagöz and Canlı 2024).

### 2.1. Participants

This study employed a three-stage sequential design involving distinct participant groups recruited from multiple teacher education faculties across Turkey during the fall semester of the 2024–2025 academic year. The independent sampling across different institutions for each stage followed best practices in scale development and validation (DeVellis 2017; Hair et al. 2019), ensuring cross-institutional generalizability while maintaining methodological rigor through sample independence.

All participants were pre-service teachers who had participated in extracurricular activities for at least one academic semester, including university-organized conferences, workshops, professional development sessions, student organizations, and academic competitions. This criterion ensured experiential familiarity with the constructs being measured. Participation was voluntary with informed consent obtained from all participants. Ethical approval was obtained from the institutional review board (Ethics Committee Decision No. GO 2024/802, 3 December 2024), with this study conducted in accordance with the Declaration of Helsinki.

Stage 1 involved 989 pre-service teachers from four teacher education faculties for exploratory factor analysis. After removing 34 participants due to univariate outliers (z-scores > 3.29), the final EFA sample comprised 955 participants (77.5% female, 22.5% male) representing first- through fourth-year students from geographically diverse regions.

Stage 2 employed an independent sample of 864 pre-service teachers recruited from four different teacher education faculties for confirmatory factor analysis. Following outlier removal (N = 48), the final CFA sample included 816 participants (79.1% female, 20.9% male) representing first- through fourth-year students with complete sample independence from Stage 1.

Stage 3 constituted the primary analysis sample of 775 pre-service teachers from a single teacher education faculty, limited to second- through fourth-year students to ensure valid GPA-based academic achievement measures. The sample included 71.1% female (N = 551) and 28.9% male (N = 224) participants distributed across academic levels: second-year students 41.8% (N = 324), third-year students 27.9% (N = 216), and fourth-year students 30.3% (N = 235). Program area distribution included Science and Mathematics Education 16.3% (N = 126), English Language Teaching 15.7% (N = 122), Special Education 14.7% (N = 114), Psychological Counseling and Guidance 6.3% (N = 49), Art and Music Education 3.5% (N = 27), Elementary Education 23.6% (N = 183), and Turkish and Social Sciences Education 19.9% (N = 154).

### 2.2. Data Collection Tool and Data Collection Process

The data were collected with the Scale for the Contribution of Participation in Extracurricular Activities to Professional Development. Academic achievement was measured using participants’ cumulative grade point averages (GPAs) obtained from university records. The scale was developed by the researcher following Price’s (2016) stages. Figure 6 shows the stages of scale development. Exploratory factor analysis (EFA) and confirmatory factor analysis (CFA) were conducted to examine the construct validity of the scale. Average variance extracted (AVE) values were calculated for the convergent validity of this study. Within the scope of reliability studies, Cronbach’s alpha reliability coefficients for internal consistency reliability and C.R. values for composite reliability were calculated.

#### Scale for the Contribution of Participation in Extracurricular Activities to Professional Development

**Figure 7:**
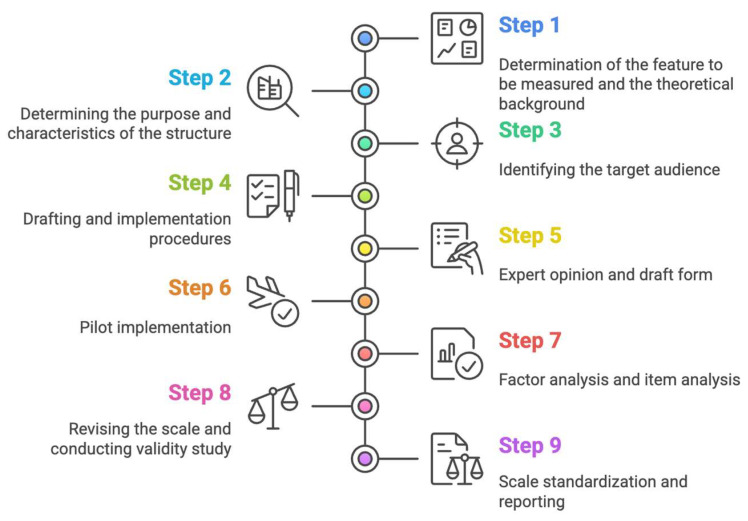
Scale development process.

In the process of developing the scale, the stages shown in Figure 7 were followed.

As seen in Figure 7, the scale development followed a systematic nine-stage process. After establishing the theoretical framework and determining target characteristics (Steps 1–3), 64 items were developed based on the theoretical frameworks (Step 4). Expert evaluation involving six specialists resulted in the removal of 4 items and revision of 4 others, creating a 60-item pilot version (Step 5). Prior to pilot implementation, participants were provided with a clear definition of extracurricular activities. The pilot study was conducted with 989 pre-service teachers (Step 6), followed by exploratory factor analysis and item analysis which resulted in the removal of 12 items due to cross-loadings (Step 7). The final 48-item, five-factor structure was then validated through confirmatory factor analysis on an independent sample of 864 pre-service teachers (Step 8), completing the standardization and reporting process (Step 9).

As seen in Figure 7, the scale items were developed to measure constructs that are compatible with the hypotheses of the study by utilizing Social Cognitive Theory, Self-Determination Theory, Self-Regulation Theory, and Interpersonal Relationships Theory. In this context, the items were written for participation in extracurricular activities, contribution of these activities to self-efficacy toward teaching, contribution to professional interest in teaching, contribution to self-regulation skills, and contribution to teacher–student relationships. The items in the “Participation in Extracurricular Activities” dimension were associated with the social learning and modeling processes in Bandura’s (1986) Social Cognitive Theory (e.g., “Extracurricular activities enhance my teaching skills through observation and practice”) and with the concepts of intrinsic motivation and autonomy in Ryan and Deci’s (2000) Self-Determination Theory. The dimension “Contribution to Self-Efficacy Toward Teaching” was structured on the basis of Bandura’s (1997) concept of self-efficacy (e.g., “Extracurricular activities help me develop effective classroom management skills”). The dimension “Contribution to Interest in the Teaching Profession” reflects the constructs of interest and intrinsic motivation in Ryan and Deci’s (2017) Self-Determination Theory (for example, “Participation in extracurricular activities increases my intrinsic interest in the teaching profession”). The “Contribution to Self-Regulation Skills” dimension includes the planning, monitoring, and evaluation processes of Zimmerman’s (2000) Self-Regulation Theory (e.g., “Through extracurricular activities, I can effectively plan and manage my study time”). The “Contribution to Teacher–Student Relationships” dimension is based on the interaction and communication quality constructs of Reis and Shaver’s (1988) Interpersonal Relationships Theory (e.g., “Extracurricular activities allow me to develop supportive relationships with faculty members”). The integrated use of these theoretical frameworks strengthened the theoretical consistency and construct validity of the scale items.

Prior to data collection, participants were provided with a clear definition of extracurricular activities: “University-organized conferences, workshops, professional development sessions, student organizations, and academic competitions that occur beyond the formal curriculum.” This definition ensured consistent understanding across all participants.

In order to assess the content validity of the scale, three subject matter experts and three measurement and evaluation experts were consulted. The experts evaluated the 64-item draft scale using a three-point rating system with the options “keep”, “modify”, and “remove”. Gwet’s AC1 coefficient (Gwet 2008) was used to determine the agreement between the experts because it controls the chance factor better. Gwet’s AC1 coefficient calculated as a result of the expert evaluations was 0.94 for subject matter experts, 0.95 for assessment experts, and 0.95 for all experts. These values are at the level of “very good agreement” according to Landis and Koch’s (1977) classification. In the evaluations, full consensus was achieved in 56 of the 64 items (87.5%). Based on the expert evaluations, 4 items were removed from the scale due to either receiving removal recommendations from at least two experts or having contradictory expert opinions. A further 4 items were revised according to expert suggestions. This process resulted in the final 60-item version of the scale. The trial form was applied to two different sample groups. The first group consisted of 989 pre-service teachers. EFA and reliability analyses were conducted on the data obtained from this group for construct validity. EFA and reliability analyses were conducted with the data obtained from 955 pre-service teachers after removing 34 univariate outliers. In total, 77.5% (N = 740) of the pre-service teachers were female and 22.5% (N = 215) were male. Principal components analysis with varimax rotation method was used as the extraction method in EFA to extract factors and determine the underlying factor structure of the scale items. Varimax rotation was applied to achieve simple structure and enhance interpretability of the factor loadings. As part of the reliability study, Cronbach’s alpha coefficient and C.R. values were calculated. Reliability and validity findings are presented in Table 1.

All scale items were originally developed in Turkish by the researcher, grounded in the theoretical frameworks of Social Cognitive Theory, Self-Determination Theory, Self-Regulation Theory, and Interpersonal Relationships Theory. Table 1 presents item content descriptions (rather than direct translations) to maintain conceptual accuracy while providing clarity about the constructs measured.

As shown in Table 1, exploratory factor analysis (EFA) was conducted to determine the factor structure of the scale. The EFA resulted in a five-factor solution. These five dimensions (Extracurricular Activities Participation [16 items], Self-Efficacy [9 items], Interest [8 items], Self-Regulation [7 items], and Relationships [8 items]) consist of a total of 48 items. Twelve items were removed from the analysis due to cross-loadings on multiple factors. The factor loadings of the remaining items were at satisfactory levels (ranging from 0.549 to 0.851), and all loadings were statistically significant (*p* < 0.05). The internal consistency reliability (Cronbach’s α) of the dimensions was found to be high (ranging from 0.91 to 0.93). Additionally, composite reliability (CR) values (0.816 to 0.912) exceeded the recommended threshold (.70) (Hair et al. 2019). The average variance extracted (AVE) values (0.521 to 0.612) indicated that convergent validity was established (Fornell and Larcker 1981). These findings support that the five-factor structure of the developed scale is psychometrically robust and valid.

Whether the five-factor structure of the scale was tested for confirmation on the data obtained from a second sample group. The second group consisted of 864 pre-service teachers. CFA was conducted on the data obtained from this group for construct validity. After removing 48 univariate outliers, CFA was conducted with the data obtained from 816 pre-service teachers. In total, 79.1% (N = 645) of the pre-service teachers were female and 20.1% (N = 171) were male.

The confirmatory factor analysis confirmed the five-factor structure with good model fit indices (χ^2^/df = 2.45, RMSEA = 0.038, CFI = 0.96, TLI = 0.95, GFI = 0.87, AGFI = 0.85), demonstrating that the scale’s construct validity was successfully established. Additionally, composite reliability (CR) exceeded 0.70 and average variance extracted (AVE) exceeded 0.50 for all constructs, further confirming the scale’s psychometric adequacy. The detailed results of the measurement model are presented in Section 3.1.

### 2.3. Data Analysis

The SPSS 20.0 package program was used to test the normality of the data, demographic characteristics of the participants, exploratory factor analysis, and Cronbach’s alpha reliability coefficients of the scale. In this study, it was determined that the kurtosis and skewness values of the subdimensions were within the −2, +2 value range, so the distribution of the scores showed a normal distribution (George 2011).

Structural equation modeling (SEM) was used to test the measurement model of the research and then the hypotheses of the research. The AMOS 22.0 (Analysis of Moment Structures) package program was used for all structural equation modeling procedures, as follows: (1) confirmatory factor analysis to test the measurement model fit, (2) assessment of measurement model validity and reliability, (3) testing the hypothesized structural relationships between latent variables, and (4) calculation of model fit indices (χ^2^/df, RMSEA, CFI, TLI, GFI, AGFI). The software was specifically chosen for its robust maximum likelihood estimation procedures and comprehensive fit statistics suitable for complex structural models.

The two-stage method proposed by Anderson and Gerbing (1984) was used in the study. Accordingly, the measurement model of the research was tested first and the established model was tested in the second stage. The validity of the scales is tested by testing the measurement model. Factor loadings, C.R. values for composite reliability and average variance extracted (AVE) values for convergent validity were calculated. While C.R. values above 0.70 are recommended, values above 0.50 are also accepted. The AVE value is recommended to be above 0.50 (Hair et al. 2021). The χ2/df ratio (CHMIN/df), comparative fit index (CFI), Tucker–Lewis index (TLI), root mean square error of approximation (RMSEA), and GFI and AGFI values were used to interpret the findings. A χ2/df value ≤ 2, RMSEA value < 0.05, AGFI ≥ 0.90, GFI ≥ 0.95, and CFI and TLI ≥ 0.97 indicate a good fit between the model and data (Hair et al. 2019). A χ2/df value ≤ 3 (Kline 2023), RMSEA value ≤ 0.08 (Byrne 2010), GFI, and CFI and TLI ≥ 0.90 (Bentler 1990) indicate that the model–data fit is acceptable. According to Anderson and Gerbing (1984), GFI ≥ 0.85 and AGFI ≥ 0.85 are also acceptable lower limits.

Measurement of model validity was assessed using confirmatory factor analysis following Anderson and Gerbing’s (1984) two-stage approach. Model fit was evaluated using multiple indices as recommended by the methodological literature: χ^2^/df ratio, RMSEA, CFI, TLI, GFI, and AGFI (Hair et al. 2019; Hu and Bentler 1999). Composite reliability and average variance extracted values were calculated to assess construct reliability and convergent validity respectively.

## 3. Results

The findings are analyzed under three main headings: findings related to the measurement model, findings related to the structural equation model, and the goodness of fit values of the model.

### 3.1. Results Related to the Measurement Model

In the first step of the structural equation modeling approach, CFA was applied to test the measurement model and confirm the five-factor structure of the instrument. As a result of CFA, χ^2^/df = 2.45, RMSEA = 0.038, CFI = 0.96, TLI = 0.95, GFI = 0.87, and AGFI = 0.85. These values indicate that the model fit the data well and the five-factor structure of the instrument was confirmed. For all constructs, the composite reliability (CR) value exceeded 0.70 and the average variance extracted (AVE) value exceeded 0.50. These values are given in Table 1. The measurement model demonstrated robust psychometric properties across all constructs. Factor loadings ranged from 0.549 to 0.851, with the majority exceeding acceptable thresholds. Composite reliability values (0.816 to 0.912) substantially exceeded the recommended 0.70 threshold, indicating strong internal consistency. Average variance extracted values (0.521 to 0.612) met the 0.50 criterion for all constructs, confirming adequate convergent validity. The confirmatory factor analysis results validated the theoretical five-factor structure, providing a solid foundation for subsequent structural model testing.

### 3.2. Goodness of Fit Values of the Model

Table 2 presents the goodness of fit values of the structural model.

When examining the goodness of fit values for the model provided in Table 2, all indices meet established acceptable thresholds: χ^2^/df = 2.855 (≤3.0; Kline 2023), RMSEA = 0.049 (≤0.06 for close fit; Hu and Bentler 1999), CFI = 0.93 (≥0.90 for acceptable fit; Hu and Bentler 1999), TLI = 0.92 (≥0.90 for acceptable fit; Hu and Bentler 1999), GFI = 0.85 (≥0.85; Hair et al. 2019), and AGFI = 0.84 (≥0.80 for acceptable fit; Hair et al. 2019; Byrne 2010). These findings indicate that the model is compatible with the data and demonstrates adequate fit. 

### 3.3. Results Related to the Structural Model

Table 3 presents the standardized regression coefficients (β), critical ratio (C.R.), multiple coefficient of determination (R^2^), and significance levels (*p*) for the structural model. In line with the theoretical framework, the relationships between variables are analyzed under three subheadings.

**Figure 8:**
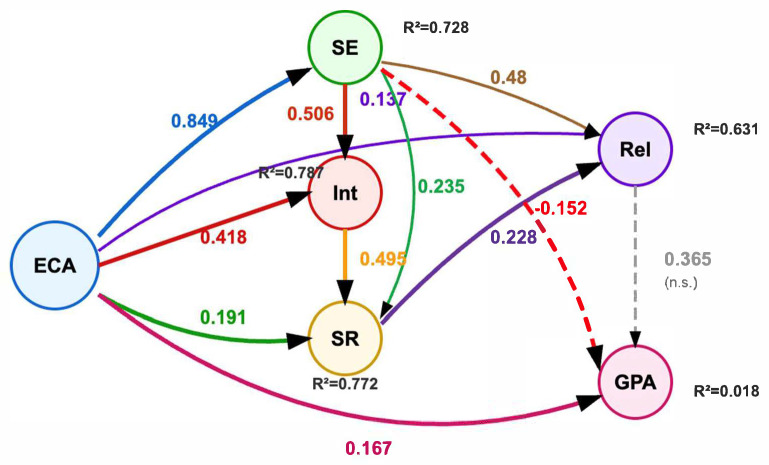
The model with standardized coefficients.

Figure 8 presents the comprehensive structural equation model with standardized coefficients, illustrating the complex network of relationships among all study variables. The model integrates findings from all three theoretical frameworks, displaying both direct effects and mediation pathways with clear visual distinctions between positive significant relationships (solid black arrows), negative significant relationships (red dashed arrows), and non-significant pathways (gray dashed arrows).

The enhanced model visualization clearly demonstrates the theoretical complexity underlying pre-service teacher professional development. Notably, the figure reveals three distinct relationship patterns: (1) strong positive associations between extracurricular activities and professional development constructs (β = 0.849 for self-efficacy, β = 0.418 for professional interest), (2) significant mediation pathways through self-efficacy and professional interest, and (3) the counterintuitive negative relationship between self-efficacy and academic achievement (β = −0.152, shown in red).

#### 3.3.1. Multiple Effects of Extracurricular Activities from the Perspective of Social Cognitive Theory

Within the framework of Social Cognitive Theory, the research findings showed that participation in extracurricular activities positively affected pre-service teachers’ self-efficacy (H_1_; β = 0.849, *p* < 0.05). Participation in extracurricular activities explained 72.8% of the variability in pre-service teachers’ self-efficacy. Similarly, in line with the prediction of the theory, it was found that participation in extracurricular activities positively affected pre-service teachers’ professional interest in teaching (H_2_; β = 0.418, *p* < 0.05).

Participation in extracurricular activities positively affected pre-service teachers’ self-regulation skills (H_3_; β = 0.191, *p* < 0.05) and teacher–student relationships (H_4_; β = 0.137, *p* < 0.05). Moreover, in line with the theoretical expectations, participation in extracurricular activities positively affected pre-service teachers’ academic averages (H_5_; β = 0.1.67, *p* < 0.05).

#### 3.3.2. Self-Efficacy, Professional Interest, and Self-Regulation Relationships from the Perspective of Self-Determination Theory

When analyzed within the framework of Self-Determination Theory, it was determined that self-efficacy positively affected professional interest in teaching (H_6_; β = 0.506, *p* < 0.05) and professional interest in teaching positively affected self-regulation skills (H_9_; β = 0.495, *p* < 0.05). Participation in extracurricular activities and self-efficacy together explained 78.7% of the variability in pre-service teachers’ professional interest in teaching. This finding shows both the direct effect of extracurricular activities and the indirect effect through self-efficacy in the development of pre-service teachers’ professional interests.

#### 3.3.3. Relational Mechanisms from the Perspective of Self-Regulation and Interpersonal Relationships Theories

From the perspective of the Self-Regulation and Interpersonal Relationships Theories, self-efficacy positively affected self-regulation skills (H_7_; β = 0.235, *p* < 0.001) and improved self-regulation skills positively affected teacher–student relationships (H_8_; β = 0.228, *p* < 0.05). In addition, self-efficacy had a direct positive effect on teacher–student relationships (β = 0.48, *p* < 0.05).

Participation in extracurricular activities, self-efficacy, and self-regulation skills together explained 63.1% of the variability in teacher–student relationships. This rate shows that the variables in the model have a strong explanatory effect on the development of teacher–student relationships.

Participation in extracurricular activities, self-efficacy, and interest together explained 77.2% of the variability in pre-service teachers’ self-regulation skills. This high rate emphasizes the importance of both the direct effects of extracurricular activities and the indirect effects of self-efficacy and professional interest in the development of self-regulation skills.

One of the most striking findings of this study is that self-efficacy has an unexpectedly negative effect on academic average (H_10_; β = −0.1.52, *p* = 0.022). This finding suggests that high self-efficacy may negatively affect academic performance in some situations. On the other hand, teacher–student relationships did not significantly predict pre-service teachers’ academic averages (H_10_; β = 0.365, *p* = 0.474); therefore, the theoretical expectation regarding the mediating role of teacher–student relationships was not supported.

Participation in extracurricular activities, relationship, and self-efficacy together explained only 1.8% of the variability in pre-service teachers’ academic averages. This low explained variance ratio indicates that other factors other than the variables in the model play an important role in the formation of academic achievement.

## 4. Discussion

In the discussion, firstly, the results are discussed by taking into account the theoretical background of the research; then, the original findings and theoretical implications, the contributions of the research in the context of the Sustainable Development Goals, and the limitations of the research are given. This discussion examines findings through the established informal learning lens, where professional development occurs through experience, reflection, and social interaction in contexts beyond formal curriculum (Eraut 2004; Marsick and Watkins 2001). Extracurricular activities represent authentic informal learning environments that align with contemporary understanding of workplace learning and professional development (Billett 2004).

### 4.1. Discussion of Multiple Effects of Extracurricular Activities from the Perspective of Social Cognitive Theory

Consistent with informal learning theory, extracurricular activities provide authentic experiential learning contexts where knowledge emerges from practice and social interaction (Eraut 2004). These settings exemplify what Wenger (1998) describes as communities of practice, where learning occurs through participation in professional activities beyond formal instruction. The strong positive effect of participation in extracurricular activities on pre-service teachers’ self-efficacy beliefs (β = 0.849) is consistent with the basic assumptions of Social Cognitive Theory. According to Bandura (1997), self-efficacy beliefs are fed from sources such as successful experiences, observational learning, social persuasion, and psychological states. Extracurricular activities provide pre-service teachers with experiences that enrich these sources and are thereby associated with improved professional skills and strengthening their self-efficacy beliefs. These findings support Kanar and Bouckenooghe’s (2021) assertion that teacher self-efficacy is strengthened by professional experiences and confirm the theoretical expectation that extracurricular activities are positively associated with self-efficacy.

Recent research further validates this theoretical prediction. Pang et al. (2025) demonstrated a significant positive correlation between active participation in career education and academic self-efficacy, while Songhori and Mehr (2025) revealed substantial relationships between teachers’ self-efficacy and work engagement. Additionally, Forneris et al. (2014) found that extracurricular participation leads to improved coping efficacy and resilience among adolescents. However, contextual factors should be considered, as Tschannen-Moran and Hoy (2001) noted that efficacy measures can vary across institutional contexts, and Klassen et al. (2020) demonstrated considerable variability in experience–efficacy relationships across different teacher education settings.

The significant effect of extracurricular activities on professional interest in teaching (β = 0.418) is an expected result within the framework of Social Cognitive Theory. As emphasized in Ginosyan et al.’s (2020) study, extracurricular activities are associated with increased motivation in pre-service teachers and deepen their professional understanding. These findings confirm the theoretical prediction that extracurricular activities are associated with increased professional interest.

The findings of this study showed that extracurricular activities had a significant effect on self-regulation skills (β = 0.191). From the perspective of Social Cognitive Theory, this relationship demonstrates how unstructured learning environments contribute to the development of self-regulation skills (Dignath and Büttner 2018). As emphasized by Zimmerman and Schunk (2013), individuals’ ability to plan, monitor, and evaluate their own learning processes develops in such environments. These findings confirm the theoretical expectation that extracurricular activities are associated with self-regulation skills.

The effect of extracurricular activities on teacher–student relationships (β = 0.137) can be explained by the critical role of social interactions in learning and development (Wang et al. 2024). These activities provide pre-service teachers with the opportunity to practice interacting with different groups of students and influence the quality of future teacher–student relationships. These findings support the theoretical expectation that extracurricular activities are positively associated with teacher–student relationships.

The positive association between extracurricular activities on academic achievement (β = 0.167) aligns with Social Cognitive Theory’s assumption that academic achievement relates to cognitive abilities, self-efficacy beliefs, and social interactions (Bandura 1997). As Zander et al. (2018) emphasized, academic and social networks enhance students’ academic integration and performance. These findings confirm the theoretical prediction that there will be a positive relationship between extracurricular activities and academic achievement.

### 4.2. Discussion of Self-Efficacy, Professional Interest, and Self-Regulation Relationships from the Perspective of Self-Determination Theory

From an informal learning perspective, the relationships between self-efficacy, interest, and self-regulation align with Billett’s (2001) assertion that workplace learning is driven by individual agency within supportive social environments. Extracurricular activities create conditions where autonomous learning naturally supports intrinsic motivation. The findings of this study showed that self-efficacy had a significant effect on interest in the teaching profession (β = 0.506). This finding is consistent with Self-Determination Theory’s (Deci and Ryan 2000) explanations about the relationship between intrinsic motivation and fulfillment of the need for efficacy. Pre-service teachers with a strong sense of efficacy show higher levels of interest and motivation towards the profession. As stated in Kaleli’s (2020) studies, pre-service teachers with high self-efficacy beliefs exhibit more positive attitudes towards the profession. These findings confirm the theoretical expectation that high self-efficacy is associated with increased professional interest.

The relationship between professional interest and self-regulation also deserves examination. A significant relationship (β = 0.495) was found between professional interest in teaching and self-regulation skills. In terms of Self-Determination Theory, the relationship between professional interest and self-regulation is explained by intrinsic motivation and autonomous learning processes (Vansteenkiste et al. 2020). As emphasized in Tikhomirova et al.’s (2021) study, strong professional interest is positively related to the use of effective self-regulation strategies. These findings support the theoretical expectation that strong professional interest will be associated with effective self-regulation skills.

Contemporary studies provide additional nuanced perspectives on this relationship. Fleet et al. (2025) and El Fadely et al. (2025) emphasize the role of intrinsic motivation in fostering self-regulation within Self-Determination Theory contexts. Liu et al. (2025) demonstrated that self-regulated learning is essential across educational contexts, with self-directed learners being more effective in achieving educational goals. Calvo et al. (2025) found that service-learning experiences contribute to autonomous deep learning necessary for self-regulation development. However, theoretical models suggest this relationship may be more complex, as Schiefele (2009) argued that interest–regulation relationships may transcend linear models, while Hidi and Renninger (2006) indicate that interest development follows distinct phases, suggesting this relationship might vary depending on which phase of interest development pre-service teachers are experiencing.

While this study demonstrates significant relationships between self-efficacy, professional interest, and self-regulation within the Self-Determination Theory framework, it is important to acknowledge both the contributions and limitations of the theoretical integration. The strong relationships observed between self-efficacy and professional interest (β = 0.506) and between professional interest and self-regulation (β = 0.495) suggest that extracurricular activities may facilitate competence need satisfaction through enhanced self-efficacy, which then supports autonomous motivation and self-directed learning behaviors.

However, the approach of examining academic achievement through Social Cognitive Theory (H_5_) and Self-Regulation Theory (H_10_) frameworks, rather than incorporating it into SDT-derived hypotheses, represents both a methodological choice and a theoretical limitation. This decision was based on conceptualizing academic achievement as a performance outcome influenced by multiple factors beyond intrinsic motivation alone. While this approach allowed the examination of achievement from multiple theoretical perspectives, it may have limited the ability to fully capture the motivational mechanisms that Self-Determination Theory proposes for performance outcomes, representing a missed opportunity to demonstrate SDT’s explanatory power for achievement outcomes in teacher education contexts.

### 4.3. Discussion of Relational Mechanisms from the Perspective of Self-Regulation and Interpersonal Relationships Theories

The relational dimension of professional development is particularly evident in informal learning contexts, where interpersonal interactions and social learning processes are central (Billett 2006). Extracurricular activities, as informal learning environments, naturally foster the kind of mentor–mentee relationships and peer interactions that support both self-regulation and relationship-building skills. The effect of self-efficacy on self-regulation skills (β = 0.235) is consistent with the basic assumptions of Self-Regulation Theory. As Panadero et al. (2017) emphasized in their study, self-efficacy beliefs play an important role in the development of self-regulation skills. According to the findings of Li and Zheng (2018), students with high self-efficacy use more effective self-regulation strategies. These findings confirm the theoretical expectation that high self-efficacy is positively associated with self-regulation skills.

The positive effect of self-regulation skills on teacher–student relationships (β = 0.228) is consistent with the predictions of Interpersonal Relationships Theory (Reis and Shaver 1988). According to this theory, regulation of emotions, openness, and sensitivity in interpersonal relationships determine the quality of relationships. As stated in Aldrup et al.’s (2018) study, pre-service teachers with strong self-regulation skills communicate more effectively with students and develop more supportive relationships. This finding is further supported by recent research from Lasekan et al. (2025), which demonstrates that pre-service teachers with strong self-regulation skills are more effective communicators, thereby fostering supportive teacher–student relationships. Additionally, Calandri et al. (2025) observed that teachers’ emotional regulation capabilities positively impact relationships with students and enhance classroom management. These findings support the theoretical expectation that improved self-regulation skills are positively associated with teacher–student relationships.

The lack of a significant effect of teacher–student relationships on academic achievement (β = 0.365, *p* > 0.05) indicates that the theoretical expectation that teacher–student relationships would mediate the relationship between self-efficacy and academic achievement was not supported. This unexpected result contradicts the study of Lei et al. (2018). This may be explained by the fact that the effects of teacher–student relationships on academic achievement could not be fully observed due to the fact that pre-service teachers have not yet started their profession. However, considering the limited power of the model to explain 1.8% of the variance in academic achievement, this result suggests that different variables should be included in future studies.

While this study demonstrates significant relationships between self-efficacy, self-regulation, and interpersonal relationships, it is important to acknowledge that self-regulation development involves multiple mechanisms beyond self-efficacy. Goal-setting strategies, metacognitive awareness, environmental factors, and motivational orientations all play crucial roles in fostering effective self-regulation (Zimmerman and Moylan 2009). For instance, structured goal-setting activities within extracurricular contexts may provide specific frameworks for developing self-regulatory competencies, while supportive environmental conditions such as peer collaboration and mentor feedback can facilitate the acquisition of self-monitoring and self-evaluation skills. Future research should explore these additional mechanisms to develop a more comprehensive understanding of how different components of extracurricular experiences contribute to self-regulation development in pre-service teachers. Such investigations could inform the design of more targeted interventions that optimize the self-regulatory benefits of extracurricular participation.

### 4.4. Original Results and Theoretical Implications

One of the most striking original findings of our study is the negative relationship between self-efficacy and academic achievement (β = −0.152, *p* < 0.05). This finding contradicts the general predictions of Social Cognitive Theory (Bandura 1997) and previous studies (Schneider and Preckel 2017) that found a positive relationship between self-efficacy and academic achievement. As Bandura (1997) emphasized, self-efficacy is generally positively related to performance in a particular domain. However, our findings suggest that this relationship may be more complex in the context of teacher education. While the model has a high explanatory power (63.1% to 78.7%) for the other variables, it has a very low explanatory power (1.8%) for the variance in academic achievement. This low rate requires caution in interpreting this relationship. This negative relationship may be explained by the fact that pre-service teachers with high self-efficacy reduce their study efforts due to overconfidence (Gebresilase and Zhao 2023) or direct themselves to practical skills rather than academic performance. Zimmerman (2000) stated that profession-specific efficacy perceptions can lead to different behavior patterns. Pre-service teachers who are developing high self-efficacy beliefs towards the teaching profession may devote more time and energy to practical applications and extracurricular professional development activities instead of focusing on theoretical courses.

However, this counterintuitive finding may reflect fundamental differences between the competencies developed through extracurricular activities and those measured by traditional academic assessments. The substantial explanatory power of our model for professional development variables (63.1–78.7%) compared to academic achievement (1.8%) suggests these represent distinct developmental domains. Extracurricular activities may enhance practical teaching competencies that are not captured by GPA-based measures, indicating potential misalignment between assessment systems and the multidimensional nature of teacher preparation.

Another unique finding was that the theoretical expectation that teacher–student relationships would mediate the relationship between self-efficacy and academic achievement was not supported. The fact that teacher–student relationships did not significantly predict academic average (β = 0.365, *p* > 0.05) suggests that other factors not addressed in the model may be more determinative in the formation of academic achievement. The limited power of the model to explain the variance in academic achievement (1.8%) suggests that variables such as cognitive abilities, study strategies, and motivational factors should be included in the model in future research. Furthermore, from the perspective of Interpersonal Relationships Theory (Reis and Shaver 1988), interpersonal relationships may play a mediating role in psychological processes. According to Reis and Shaver’s (1988) model, interaction processes need to be mature enough for the mediation effect of interpersonal relationships to develop. The fact that pre-service teachers have not yet started their profession may indicate that the mediating role of these relationships may not be fully developed.

Contemporary research provides additional insights into this complex relationship. Zeb et al. (2025) suggest that the limited mediating effect may indicate that other factors are more impactful in determining academic success, emphasizing the need to explore cognitive abilities, study strategies, and motivational factors. From the perspective of Interpersonal Relationships Theory, Zhao et al. (2025) support the notion that the professional inexperience of pre-service teachers may mean these relationships are not sufficiently developed to exert anticipated mediating effects. Importantly, Zhao et al. (2025) also highlight that self-efficacy can moderate the effects of stress on academic performance, suggesting that self-efficacy’s robust influence may overshadow relationship factors. Xiang et al. (2025) further emphasizes that while teacher–student relationships are significant, broader support systems including family and peer dynamics may be more influential in academic achievement, suggesting future research should incorporate a more comprehensive conceptualization of educational relationships.

The strong effect of extracurricular activities on self-efficacy (β = 0.849) confirms the thesis of Social Cognitive Theory (Bandura 1997) regarding the critical role of direct experience in self-efficacy development. This effect has important reflections in the international teacher education literature. Darling-Hammond (2017) stated that effective teacher education programs should emphasize practical experiences as well as academic knowledge. Our findings suggest that teacher evaluation approaches based only on academic achievement may be insufficient to measure all dimensions of teacher competencies. Furthermore, it is important to acknowledge that contextual factors may influence the relationships observed in this study. Institutional characteristics such as urban versus rural settings, public versus private institutions, available resources, and cultural contexts may moderate the effectiveness of extracurricular activities in supporting pre-service teacher development. For instance, urban institutions may offer more diverse extracurricular opportunities, while rural settings might provide different types of community engagement experiences. These contextual variations should be considered when interpreting findings and represent important avenues for future research examining the generalizability of these relationships across different educational contexts.

From the perspective of Self-Determination Theory (Deci and Ryan 2000), the effect of extracurricular activities on professional interest (β = 0.418) can be explained by intrinsic motivation and autonomy. According to Deci and Ryan (2000), meeting the needs for autonomy, competence, and relationship increases intrinsic motivation. Extracurricular activities can increase pre-service teachers’ professional interest by providing them with the opportunity to meet these basic psychological needs. A longitudinal study by Mahoney et al. (2003) showed that organized extracurricular activities positively affect the development of young individuals and that this effect is long-lasting.

From the perspective of Self-Regulation Theory (Zimmerman 2000), the effect of extracurricular activities on self-regulation skills (β = 0.191) is related to the development of individuals’ capacity to regulate their learning processes. Zimmerman (2008) emphasized the importance of developing self-regulation skills in informal learning environments.

While these findings provide valuable insights into extracurricular activities’ associations with professional development, it is important to acknowledge that the relationships observed may not represent direct causal impacts but rather complex associations mediated by multiple factors (Hayes 2017). Several moderating variables could influence these outcomes, including participants’ previous teaching experiences (such as tutoring or classroom volunteer work), socioeconomic backgrounds that may affect access to and motivation for extracurricular participation (Pascarella and Terenzini 2005), and cultural backgrounds that shape perceptions of professional development activities (Sue et al. 2019). Additionally, different types of extracurricular activities may contribute differentially to teacher competencies (Kuh 2008). Academic-oriented activities (such as research projects or subject-specific competitions) may particularly enhance content knowledge and pedagogical skills, while social activities (such as student organizations or peer mentoring) may primarily develop interpersonal and leadership competencies. Cultural activities (including cultural events or community service) may foster cultural competence and social awareness essential for diverse classroom environments (Banks 2015). Future research should examine these activity-specific effects and potential moderating variables to develop a more nuanced understanding of how different forms of extracurricular engagement contribute to professional development outcomes.

These findings collectively demonstrate that extracurricular activities function as effective informal learning environments, validating the theoretical premise established in this study. The complex relationships observed align with Fenwick’s (2008) assertion that informal learning in professional contexts involves dynamic interactions between individual agency, social relationships, and authentic practice contexts.

### 4.5. Contributions to Sustainable Development Goal 4 (Quality Education)

This research contributes to SDG 4 by demonstrating how extracurricular activities are associated with enhanced teacher preparation quality through improved self-efficacy, professional interest, and teacher–student relationships. The findings align with UNESCO’s (2020) framework for Education for Sustainable Development.

Extracurricular activities help pre-service teachers develop sustainability competencies including systems thinking, anticipatory thinking, normative competency, strategic thinking, collaboration, critical thinking, self-awareness, and integrated problem-solving skills (UNESCO 2020). These competencies are essential for preparing students for complex 21st century challenges. Notably, the negative relationship between self-efficacy and academic achievement (β = −0.152) suggests that teacher evaluation frameworks should incorporate multiple competency dimensions rather than relying solely on academic performance measures.

### 4.6. Integration of Formal and Informal Learning in Teacher Professional Development

This study’s findings illuminate the complementary nature of formal and informal learning components in teacher professional development, with important implications for understanding how different learning contexts contribute to educator effectiveness. Teacher professional development encompasses multiple interconnected components including pedagogical content knowledge, instructional methodology, educational psychology, classroom management, assessment methodologies, and supervised field experiences that provide structured educational experiences through systematic curriculum frameworks (Darling-Hammond et al. 2017; Shulman 1987). While these formal curriculum components provide essential foundational knowledge through systematic instruction and structured progression pathways, the strong associations observed between extracurricular activities and professional development outcomes (self-efficacy β = 0.849, professional interest β = 0.418) demonstrate that informal learning makes substantial contributions beyond formal requirements.

The theoretical frameworks examined in this study—Social Cognitive Theory, Self-Determination Theory, and Self-Regulation Theory—operate across both formal and informal learning contexts rather than being limited to extracurricular activities alone. Social Cognitive Theory’s emphasis on observational learning and modeling occurs within formal settings through structured field experiences, mentor-guided practice sessions, and systematic feedback mechanisms that build self-efficacy through guided mastery experiences (Bandura 1997). Simultaneously, informal contexts like extracurricular activities provide opportunities for autonomous observation, spontaneous learning experiences, and self-directed modeling of professional behaviors that complement formal curriculum constraints. The strong association between extracurricular activities and self-efficacy (β = 0.849) suggests that both contexts contribute uniquely to efficacy development through different but complementary mechanisms.

Self-Determination Theory’s principles manifest distinctly across formal–informal boundaries, demonstrating the theory’s broad applicability rather than exclusive relevance to extracurricular contexts. While formal curriculum addresses autonomy through structured choices within predetermined pathways and competency-based progression systems, extracurricular activities provide genuine autonomy through voluntary participation and self-directed exploration (Ryan and Deci 2000). Research demonstrates that when professional development activities fulfill basic psychological needs of autonomy, competence, and relatedness, they significantly increase teachers’ motivation and psychological well-being across both formal and informal contexts (Pelletier and Rocchi 2016). The relationships observed between self-efficacy and professional interest (β = 0.506) and between professional interest and self-regulation (β = 0.495) illustrate how these theoretical mechanisms operate effectively in informal learning environments while complementing formal curriculum applications.

Similarly, the Self-Regulation and Interpersonal Relationships Theories function within both formal and informal contexts, each offering distinct developmental opportunities. Formal teacher education provides structured frameworks for developing self-regulation through planned reflection activities and guided metacognitive practices, while informal learning through extracurricular activities enables spontaneous self-assessment and autonomous goal-setting that cannot be fully replicated in formal academic contexts (Zimmerman 2000; Reis and Shaver 1988).

The complex relationships revealed in this study—including the unexpected negative association between self-efficacy and academic achievement (β = −0.152)—highlight the multifaceted nature of teacher development that extends beyond traditional academic metrics. This finding suggests that formal academic requirements and informal professional development may operate according to different success indicators and developmental pathways. While formal curricula emphasize knowledge acquisition and academic performance measures through standardized assessments, informal learning through extracurricular activities may prioritize practical skill application, authentic problem-solving, and professional identity development that are not captured by traditional academic assessments but may be more predictive of long-term teaching effectiveness.

The limited explanatory power for academic achievement (1.8% variance) particularly underscores the importance of formal curriculum components not addressed in this study. Variables such as systematic content mastery, pedagogical knowledge development, and structured field experience progression—all central to formal teacher education—likely contribute substantially to academic performance measures while operating through different mechanisms than those examined in this informal learning study.

### 4.7. Practical Implications for Teacher Education Programs

The findings of this study offer several actionable insights for enhancing teacher education programs through the strategic integration of extracurricular activities. These implications are organized around three key areas: curriculum integration, assessment reform, and institutional support mechanisms.

#### 4.7.1. Strategic Integration of Extracurricular Activities

The strong relationship between extracurricular participation and self-efficacy (β = 0.849) demonstrates that teacher education programs should systematically embed experiential learning opportunities within their curricula. Rather than treating extracurricular activities as optional add-ons, programs should consider them as essential components of professional development. This aligns with Darling-Hammond’s (2017) emphasis on the importance of authentic teaching experiences in developing teacher competencies. Specifically, programs could establish mandatory participation requirements in activities such as peer tutoring, educational conferences, community service projects, and collaborative research initiatives.

The positive effects on professional interest (β = 0.418) and self-regulation skills (β = 0.191) suggest that these activities should be designed to provide meaningful choices and autonomy, consistent with Self-Determination Theory principles (Ryan and Deci 2017). Teacher education programs could develop activity portfolios that allow pre-service teachers to select from diverse options based on their interests and career goals while ensuring exposure to varied educational contexts.

#### 4.7.2. Reimagining Assessment Approaches

The unexpected negative relationship between self-efficacy and academic achievement (β = −1.519) challenges traditional assessment paradigms in teacher education. This finding suggests that relying solely on GPA as an indicator of teacher readiness may be insufficient and potentially misleading. Programs should develop more comprehensive assessment frameworks that evaluate multiple dimensions of teacher competency.

Following established teacher evaluation research (Darling-Hammond and Bransford 2007), institutions could implement portfolio-based evaluation systems that document growth in self-efficacy, professional interest, and interpersonal skills alongside academic performance. This might include reflective journals documenting extracurricular experiences, peer evaluations of collaborative projects, and self-assessment tools measuring professional development across multiple domains.

The high explanatory power of the model for non-academic variables (63.1% to 78.7%) compared to academic achievement (1.8%) indicates that these alternative measures may be more predictive of future teaching effectiveness than traditional academic metrics. This aligns with research by Allen et al. (2022) on the multifaceted nature of teacher effectiveness.

#### 4.7.3. Institutional Support and Policy Development

The findings highlight the need for institutional policies that actively support and recognize extracurricular participation. This includes allocating resources for activity coordination, providing faculty incentives for mentoring extracurricular initiatives, and establishing partnerships with schools and community organizations to create meaningful engagement opportunities.

Programs should also consider developing structured reflection processes that help pre-service teachers connect their extracurricular experiences to theoretical frameworks learned in coursework. This integration supports the development of practical wisdom, which Darling-Hammond and Bransford (2007) identify as crucial for effective teaching.

#### 4.7.4. Professional Development Pathways

The significant relationships between self-efficacy, professional interest, and self-regulation suggest that extracurricular activities could serve as vehicles for ongoing professional development. Teacher education programs could establish mentorship networks where advanced students guide newcomers through extracurricular experiences, fostering both leadership skills and peer support systems.

Additionally, the positive impact on teacher–student relationships (β = 0.137) indicates that programs should create opportunities for pre-service teachers to interact with diverse student populations through activities such as after-school tutoring, summer camps, or community education initiatives. These experiences can help develop the interpersonal competencies essential for effective teaching.

#### 4.7.5. Implementation Considerations

Successful integration of these recommendations requires careful consideration of implementation strategies. Programs should begin with pilot initiatives to test the effectiveness of specific extracurricular activities before full-scale implementation. Regular evaluation and feedback mechanisms should be established to ensure that activities are meeting their intended learning objectives.

Furthermore, faculty development may be necessary to help education professors understand how to effectively integrate and assess extracurricular experiences within their courses. This professional development should emphasize the theoretical foundations that support experiential learning and provide practical strategies for implementation.

The implications of this study extend beyond individual programs to broader policy considerations. Accreditation bodies and education departments should consider revising standards to explicitly recognize the value of extracurricular experiences in teacher preparation, potentially requiring documentation of such experiences for program approval or teacher certification.

#### 4.7.6. Addressing Contradictory Findings in Practice

The unexpected negative relationship between self-efficacy and academic achievement (β = −0.152) requires careful interpretation for practical implementation. This finding suggests that teacher education programs should adopt a nuanced approach that recognizes different types of competency development. While extracurricular activities enhance professional competencies essential for teaching effectiveness, traditional academic measures may not fully capture these benefits.

Programs should therefore implement multidimensional assessment frameworks that value both academic achievement and professional development competencies rather than viewing them as competing objectives. The high explanatory power for professional development variables (63.1% to 78.7%) compared to academic achievement (1.8%) indicates that these represent different but complementary aspects of teacher preparation that require distinct evaluation approaches. This finding suggests that academic achievement alone may not fully reflect the professional competencies developed through extracurricular engagement. The substantial variance explained in professional variables compared to academic achievement indicates that different evaluation approaches may be needed to capture the multidimensional nature of teacher preparation demonstrated in this study.

### 4.8. Measurement Model Implications

The robust psychometric properties of the developed instrument contribute significantly to teacher education assessment methodology. The five-factor structure demonstrated strong explanatory power for professional development variables (R^2^ = 63.1% to 78.7%), indicating that extracurricular activity effects can be reliably measured across multiple competency domains. However, the limited variance explanation for academic achievement (R^2^ = 1.8%) suggests that traditional academic performance measures may inadequately capture the professional competencies developed through informal learning experiences.

These measurement findings reinforce the need for multidimensional assessment approaches in teacher education that capture both academic knowledge and practical professional competencies. The substantial difference in explanatory power between professional development outcomes and academic achievement indicates that these represent distinct but complementary dimensions of teacher preparation that require different evaluation approaches.

### 4.9. Limitations of This Study

Although this study provides a comprehensive model for examining the effects of pre-service teachers’ participation in extracurricular activities on various professional development dimensions, it has several limitations. Being aware of these limitations is important for interpreting the findings and designing future research.

An important limitation of this study is its specific focus on informal learning through extracurricular activities while not systematically examining the contributions of formal curriculum components to teacher professional development. Teacher education encompasses multiple interconnected dimensions, including formal coursework in pedagogical content knowledge, instructional methodology, educational psychology, classroom management, and assessment practices. These formal curriculum components provide essential foundational knowledge, systematic skill development, and structured progression through competency frameworks that are crucial for teacher preparation.

While this study’s focus on extracurricular activities addresses a significant gap in understanding informal learning contributions to professional development, it does not capture the complex interactions between formal and informal learning components. The theoretical frameworks employed—Social Cognitive Theory, Self-Determination Theory, and Self-Regulation Theory—operate across both formal and informal contexts, yet this study examines their application specifically within extracurricular settings. Future research should investigate how these theories manifest within formal curriculum components and how formal–informal learning interactions influence overall professional development outcomes.

Additionally, several key variables related to formal curriculum remain uncontrolled in this study, including specific teacher education program structures, course sequencing, field experience requirements, and assessment methodologies. These formal components may significantly influence the effectiveness of extracurricular activities and moderate the relationships observed in this study. The limited explanatory power for academic achievement (1.8% variance explained) particularly suggests that formal academic factors not addressed in this model may be more influential in determining traditional academic outcomes than the informal learning variables examined.

This research has a cross-sectional design and data were collected in a single time period. This limits the precise identification of causal relationships between variables. For example, it was concluded that participation in extracurricular activities increases self-efficacy, but the possibility that pre-service teachers with high self-efficacy prefer to participate more in extracurricular activities should not be ignored. This selection bias extends beyond self-efficacy to include other pre-existing characteristics such as motivation levels, academic engagement patterns, socioeconomic status, and personality traits that may predispose certain individuals to seek extracurricular involvement, potentially confounding the observed relationships between participation and professional development outcomes. Therefore, the findings should be interpreted in a correlational context, and causality claims should be made with caution.

Another limitation is the use of self-report measures. Variables such as self-efficacy, professional interest, self-regulation, and teacher–student relationships were assessed using a scale reflecting participants’ own perceptions. In future research, the use of multiple data sources and measurement methods may reduce this limitation.

An additional limitation concerns validity assessment. While this study established construct validity through EFA and CFA with satisfactory convergent validity indicators, future research could strengthen methodological rigor by incorporating criterion-related validity and discriminant validity testing as suggested by the established psychometric literature (Messick 1995).

An additional methodological limitation concerns measurement invariance testing across academic levels. While our sample included students from second- through fourth-year levels, we did not assess whether the factorial structure remains invariant across these academic experience levels. Students at different stages of teacher education may have varying conceptualizations of professional development constructs and different levels of extracurricular activity experience, potentially affecting the generalizability of our findings across student cohorts (Cheung and Rensvold 2002).

Additionally, while our sample included students from second- through fourth-year levels, academic level was not included as a control variable in the structural model. Students at different stages of teacher education likely have varying baseline levels of self-efficacy, professional interest, and relationship-building experience, which may moderate the effects of extracurricular participation. The absence of academic level controls may confound the relationships observed in this study.

Additionally, this study is limited to the students of the faculty of education of a single university, which constrains generalizability across different institutional contexts. While our sample included multiple program areas (Science and Mathematics Education, English Language Teaching, Special Education, etc.), program-specific effects were not systematically controlled. Different teacher education programs may have varying emphases on extracurricular participation, distinct professional development philosophies, and different structural requirements that could moderate the relationships observed. Institutional support mechanisms, available resources, and cultural attitudes toward extracurricular activities may vary significantly across different educational contexts, potentially affecting both participation rates and developmental benefits.

While the theoretical framework is grounded in Self-Determination Theory’s three basic needs (autonomy, competence, relatedness), the measurement approach focused on outcomes related to these needs rather than directly measuring need satisfaction itself. Self-efficacy was measured as a reflection of competence experiences and professional interest as an indicator of intrinsic motivation, but participants’ sense of achievement or competence need satisfaction during extracurricular participation was not directly assessed. Additionally, academic achievement was examined through Social Cognitive Theory and Self-Regulation Theory frameworks rather than being incorporated into SDT-derived hypotheses (H_6_, H_9_), representing a missed opportunity to test whether extracurricular activities enhance academic performance through competence need satisfaction pathways. This approach may have limited the ability to comprehensively test Self-Determination Theory’s full explanatory mechanisms for achievement outcomes in teacher education contexts.

Another significant limitation is the lack of detailed information on the nature, types, and characteristics of extracurricular activities. While participation in extracurricular activities was measured as a general construct, critical factors such as activity type (academic vs. social vs. cultural), duration of participation, intensity of involvement, quality of experiences, and specific roles within activities were not examined in detail. Different types of extracurricular activities may have differential effects on professional development outcomes (Kuh 2008), and the absence of these controls limits our ability to understand which specific activity characteristics drive the observed relationships.

Another important limitation of this study is that the variables examined (participation in extracurricular activities, teacher–student relationships, and self-efficacy) can explain only 1.8% of the variance in pre-service teachers’ academic achievement. This low explained variance rate indicates that many other factors that were not addressed in this study may be effective in the formation of academic achievement. An important limitation of this study is that variables such as cognitive abilities, prior learning, study strategies, socioeconomic factors, institutional variables, and assessment practices were not included in the model. In future research, models that can explain pre-service teachers’ academic achievement more comprehensively can be tested.

Finally, measuring academic achievement only by GPA may not fully reflect the complex nature of academic achievement. More comprehensive and multidimensional measures of pre-service teachers’ learning and academic development may contribute to a more accurate understanding of the relationships between variables.

Despite these limitations, this study provides an important step forward in understanding the role of extracurricular activities in the professional development of pre-service teachers and provides guiding findings for future research.

## 5. Conclusions and Recommendations

In this study, conclusions and recommendations are given under subheadings in line with the research results.

### 5.1. Conclusions

This study examined how pre-service teachers’ participation in extracurricular activities is associated with their professional development through a comprehensive structural model grounded in Social Cognitive Theory, Self-Determination Theory, and Self-Regulation and Interpersonal Relationships Theories. A psychometrically robust five-factor scale was developed and validated, measuring extracurricular participation, self-efficacy, professional interest, self-regulation, and teacher–student relationships.

Key findings demonstrated strong associations between extracurricular activities and self-efficacy (β = 0.849), professional interest (β = 0.418), self-regulation (β = 0.191), teacher–student relationships (β = 0.137), and academic achievement (β = 0.167). The model explained substantial variance in self-efficacy (72.8%), professional interest (78.7%), self-regulation (77.2%), and teacher–student relationships (63.1%), while academic achievement showed minimal variance explanation (1.8%).

This research validates the theoretical foundation that positioned extracurricular activities as informal learning environments associated with teacher professional development. The findings demonstrate that these authentic learning contexts, characterized by experience-based learning, reflection, and social interaction (Eraut 2004; Marsick and Watkins 2001), are linked to holistic development of teaching competencies through processes consistent with informal learning theory.

A striking original finding was the negative relationship between self-efficacy and academic achievement (β = −0.152), suggesting that teacher evaluation approaches should extend beyond traditional academic metrics to include professional competencies. This unexpected result indicates the complexity of teacher development and highlights the need for holistic assessment approaches that capture the multifaceted nature of teaching effectiveness.

In conclusion, extracurricular activities are associated with strategic importance in teacher education, providing rich learning environments that are linked to holistic professional development. To realize quality education aligned with Sustainable Development Goals, teacher education programs should integrate diverse experiential learning opportunities beyond formal curriculum, recognizing that effective teacher preparation is associated with both academic knowledge and practical professional competencies developed through authentic practice contexts.

### 5.2. Recommendations

The recommendations are structured for practitioners, researchers, and policy makers in the field of teacher education and are detailed under three subheadings below.

#### 5.2.1. Recommendations for Practitioners

The research findings demonstrate that extracurricular activities explain substantial variance in professional development outcomes (63.1% to 78.7%) while showing limited association with traditional academic measures (1.8%). These results have important implications for teacher education practice:

1. Strategic integration of extracurricular activities: Programs should systematically embed experiential learning opportunities, recognizing their strong association with self-efficacy development (β = 0.849) and professional interest enhancement (β = 0.418).
2. Holistic assessment framework: The negative relationship between self-efficacy and academic achievement (β = −0.152) suggests that traditional GPA-based assessments may inadequately capture professional competencies. Programs should develop multidimensional evaluation systems incorporating professional development indicators alongside academic performance. Future research should explore assessment methodologies that align with the multidimensional nature of teacher competency development demonstrated in this study.
3. Differentiated competency recognition: The findings indicate that professional and academic competencies may develop through different pathways. Rather than viewing this as problematic, programs should recognize and nurture both dimensions as complementary aspects of teacher preparation.
4. Evidence-based resource allocation: The high explanatory power for professional development variables justifies institutional investment in extracurricular programming as a legitimate component of teacher preparation infrastructure.

#### 5.2.2. Suggestions for Researchers

The following suggestions can be given to the researchers in terms of examining the relationship between self-efficacy and academic achievement, focusing on the types of activities, enriching the components of academic achievement and types of research:

5. Examining the relationship between self-efficacy and academic achievement: It is suggested that the negative relationship between self-efficacy and academic achievement (β = −0.152) and the finding that the variables in the model explain only 1.8% of the variance in academic achievement should be examined in depth in future studies. As suggested by Schneider and Preckel (2017), research focusing on identifying additional variables such as cognitive strategies, motivational factors, socioeconomic variables, and institutional factors that can explain this paradoxical relationship and low explained variance can contribute to the field.
6. Comparative analysis of activity types and components of academic achievement: A comparative analysis of the effects of different types of extracurricular activities (academic, social, community service) on pre-service teachers’ development can be suggested. In addition, the development of comprehensive models that include cognitive, motivational, social, and institutional factors that can better explain academic achievement may help to identify the sources of the 98.2% variance that cannot be explained in the current study.
7. Longitudinal and mixed-methods research: Based on the mixed-method approaches suggested by Tashakkori and Teddlie (2010), longitudinal studies that follow the performance of pre-service teachers after they enter the profession are recommended. Designing mixed-method studies that combine qualitative and quantitative methods to understand the effects of extracurricular activities in depth can fill the gap in the literature.
8. Comprehensive Self-Determination Theory testing: Future research should address the theoretical limitations identified in this study by developing more comprehensive SDT testing approaches. The incomplete integration of Self-Determination Theory’s competence need satisfaction construct represents a significant opportunity for methodological advancement. Studies should include direct measures of basic psychological need satisfaction (autonomy, competence, relatedness) during extracurricular participation using validated instruments such as the Basic Psychological Needs Scale (Deci and Ryan 2000) or the Psychological Need Satisfaction and Frustration Scale (Chen et al. 2015). Additionally, achievement outcomes should be tested as SDT-derived hypotheses, examining pathways such as professional interest → academic achievement and competence need satisfaction → academic achievement, rather than positioning academic outcomes within alternative theoretical frameworks. Studies should also distinguish between different types of achievement experiences (mastery achievements, social recognition, goal attainment) and their differential effects on need satisfaction and subsequent professional development outcomes. Longitudinal designs would be particularly valuable for tracking need satisfaction development over time and its relationship to both immediate outcomes (motivation, engagement) and long-term professional development indicators (teaching effectiveness, career persistence).
9. Measurement invariance assessment: Future research should conduct multi-group confirmatory factor analysis to test measurement invariance (configural, metric, scalar) across academic levels and program types. This would ensure that the same constructs are being measured consistently across student cohorts with different levels of professional development experience (Widaman and Reise 1997). Such analysis would strengthen confidence in the generalizability of the scale structure across different stages of teacher education.
10. Comprehensive control variable analysis: Future research should systematically control for activity characteristics (type, duration, intensity), academic level, program type, and institutional context. Multi-level modeling approaches could account for nested data structures (students within programs within institutions) while examining how different activity types contribute differentially to specific professional development outcomes.

#### 5.2.3. Recommendations for Policymakers

The following recommendations can be made to policy makers in the context of integrated teacher training policies, financial support and competency frameworks:

11. Integrated teacher education policies: The findings suggest that in line with the United Nations’ (2015) Sustainable Development Goals, it may be beneficial to make extracurricular activities an integral part of teacher education policies. It may be suggested that Rieckmann’s (2017) sustainable development perspective should be concretely reflected in teacher education programs.
12. Financial support mechanisms: The research findings support the importance of providing systematic financial support for the organization of extracurricular activities and the participation of pre-service teachers. As Darling-Hammond et al. (2017) emphasize, considering the associations between these activities in the multidimensional development of pre-service teachers, it may be recommended to review resource allocation.
13. Updated competency frameworks: In line with the 21st century skills emphasized by the OECD (2019), teacher competency frameworks should be updated to include self-efficacy, self-regulation skills, and interpersonal relationship skills based on Reis and Shaver’s (1988) Interpersonal Relationships Theory. Expanding accreditation processes to include criteria to evaluate the quality of extracurricular activities can contribute to the quality of education.

In line with the findings of this study, these recommendations provide guidance on the strategic importance of extracurricular activities in teacher education and the support of the multidimensional development of pre-service teachers.

## Figures and Tables

**Table 1:** Exploratory factor analysis results and psychometric properties of the scale.

Dimension	Item	Item Content Description	Factor Loading	α	CR	AVE	N of Items
Extracurricular Activities Participation (ECA)	I8	Positive faculty relationship impact	0.776	0.93	0.912	0.612	16
	I7	Teaching competence enhancement	0.773				
	I6	Self-regulation contribution	0.759				
	I9	Theory-to-practice application	0.733				
	I20	Time balance management	0.732				
	I10	Collaborative motivation boost	0.729				
	I5	Professional interest enhancement	0.727				
	I4	Professional perception improvement	0.711				
	I16	Academic–extracurricular balance	0.698				
	I13	Social network expansion	0.698				
	I12	Academic growth opportunity	0.69				
	I14	Academic anxiety reduction	0.675				
	I18	University life enrichment	0.635				
	I11	University social engagement	0.624				
	I19	Social skills development	0.591				
	I21	Personal development importance	0.549				
Self-Efficacy (SE)	I29	Teaching effectiveness development	0.826	0.92	0.877	0.532	9
	I24	Classroom management skills	0.809				
	I22	Lesson planning competence	0.807				
	I26	Instructional strategy adaptation	0.802				
	I28	Student motivation capability	0.781				
	I32	Innovative teaching approaches	0.769				
	I31	Individual differences consideration	0.763				
	I30	Effective assessment skills	0.743				
	I25	Professional challenges management	0.734				
Interest (Int)	I41	Professional passion enhancement	0.851	0.92	0.875	0.533	8
	I39	Knowledge acquisition motivation	0.827				
	I42	Professional discussion enjoyment	0.817				
	I34	Current developments interest	0.812				
	I38	Professional growth excitement	0.803				
	I33	Career attractiveness perception	0.799				
	I37	Interest maintenance	0.77				
	I35	Professional interest discovery	0.745				
Self-Regulation (SR)	I48	Goal-setting for performance	0.844	0.91	0.816	0.521	7
	I46	Study motivation enhancement	0.817				
	I45	Learning process evaluation	0.81				
	I51	Regular self-assessment	0.802				
	I50	Self-regulation feedback integration	0.797				
	I49	Time management planning	0.794				
	I44	Study habits organization	0.778				
Relationships (Rel)	I54	Question-asking ability development	0.829	0.92	0.855	0.542	8
	I55	Academic support perception	0.821				
	I59	Positive relationship-building	0.816				
	I60	Effective communication development	0.806				
	I57	Developmental feedback reception	0.805				
	I58	Professional development tracking	0.801				
	I53	Supportive environment perception	0.788				
	I52	Respect and recognition	0.753				

**Table 2:** Goodness of fit values of the structural model.

Index	Value	Acceptable Fit Indicator
χ^2^/df	2.855	2 < χ^2^/df ≤ 3
RMSEA	0.049	0.05 ≤ RMSEA ≤ 0.10
CFI	0.93	0.90 ≤ CFI < 0.95
TLI	0.92	0.90 ≤ TLI < 0.95
GFI	0.85	0.85 ≤ GFI < 0.95
AGFI	0.84	0.80 ≤ AGFI < 0.90

Note: Fit index thresholds based on established criteria: χ^2^/df ≤ 3.0 indicates acceptable fit (Kline 2023); RMSEA ≤ 0.06 indicates close fit and ≤ 0.08 indicates acceptable fit (Hu and Bentler 1999); CFI ≥ 0.95 indicates excellent fit and ≥ 0.90 indicates acceptable fit (Hu and Bentler 1999); TLI ≥ 0.95 indicates excellent fit and ≥ 0.90 indicates acceptable fit (Hu and Bentler 1999); GFI ≥ 0.85 and AGFI ≥ 0.80 indicate acceptable fit (Hair et al. 2019; Byrne 2010).

**Table 3:** Structural model results.

Relationship			Theoretical Framework	Hypothesis	β	C.R.	R^2^	*p*	Result
SE	<---	ECA	Social Cognitive	H_1_	0.849	18.734	0.728	***	Supported
Int	<---	ECA	Social Cognitive	H_2_	0.418	8.301	0.787 ^a^	***	Supported
SR	<---	ECA	Social Cognitive	H_3_	0.191	3.745	0.772 ^b^	***	Supported
Rel	<---	ECA	Social Cognitive	H_4_	0.137	2.397	0.631 ^c^	0.017	Supported
GPA	<---	ECA	Social Cognitive	H_5_	0.167	2.656	0.018 ^d^	0.008	Supported
Int	<---	SE	Self-Determination	H_6_	0.506	9.78	0.787 ^a^	***	Supported
SR	<---	SE	Self-Regulation and Interpersonal Relationships	H_7_	0.235	3.963	0.772 ^b^	***	Supported
Rel	<---	SR	Self-Regulation and Interpersonal Relationships	H_8_	0.228	8.165	0.631 ^c^	***	Supported
SR	<---	Int	Self-Determination	H_9_	0.495	8.451	0.772 ^b^	***	Supported
Rel	<---	SE	Self-Regulation and Interpersonal Relationships	H_10_	0.48	3.262	0.631 ^c^	0.001	Supported
GPA	<---	Rel	Self-Regulation and Interpersonal Relationships	H_10_	0.365	0.716	0.018 ^d^	0.474	Not
									Supported
GPA	<---	SE	Self-Regulation and Interpersonal Relationships	H_10_	−0.152	−2.288	0.018 ^d^	0.022	Supported

Note: ECA: extracurricular activities, SE: self-efficacy, Int: professional interest in teaching, SR: self-regulation, Rel: teacher–student relationships, GPA: grade point average. Note 2: Symbols ^a,b,c,d^ indicate the total explained variance values for the same dependent variable. R^2^ values are as follows: ^a^ For professional interest in teaching, ECA and SE together explain 78.7%; ^b^ for self-regulation, ECA, SE, and Int together explain 77.2%; ^c^ for teacher–student relationships, ECA, SE, and SR together explain 63.1%; ^d^ for grade point average, ECA, SE, and Rel together explain 1.8% of variance. Note 3: *** *p* < 0.001.

## Data Availability

The data presented in this study are available on request from the corresponding author due to privacy and ethical considerations regarding participant information.
